# Is the incidence of meningiomas underestimated? A regional survey

**DOI:** 10.1038/sj.bjc.6604438

**Published:** 2008-06-24

**Authors:** S Larjavaara, H Haapasalo, R Sankila, P Helén, A Auvinen

**Affiliations:** 1 Tampere School of Public Health, University of Tampere, Tampere, Finland; 2 Department of Pathology, Tampere University Hospital, Tampere, Finland; 3 Unit of Neurosurgery, Tampere University Hospital, Tampere, Finland; 4 Finnish Cancer Registry, Helsinki, Finland; 5 Radiation and Nuclear Safety Authority Helsinki, Helsinki, Finland

**Keywords:** bias, incidence, meningioma, registries

## Abstract

We assessed the undercount of meningiomas in a population-based cancer registry. A comprehensive material was formed by compiling hospital sources with the Finnish Cancer Registry database. The completeness of each source ranged 62–69%. The corrected age-standardised meningioma incidence was 2.9/100 000 for men and 13.0/100 000 for women, a third higher than the cancer registry figures.

Meningiomas are typically benign tumours, arising from the meninges of the brain (in at least 90% of the cases) and the spinal cord (Berger and Prados, 2004). They are benign in more than 90% of the cases, borderline/atypical in approximately 5% of the cases and malignant in less than 5% of the cases (Claus *et al*, 2005). Meningiomas are the most frequently reported intracranial tumours, accounting for approximately one-fourth of all reported primary brain neoplasms (Surawicz *et al*, 1999; Claus *et al*, 2005).

The age-standardised (world population) national incidence rates of meningiomas reported by the Finnish Cancer Registry (FCR) are 1.6 per 100 000 for men and 5.5 for women in 2001. The corresponding rates in the Nordic countries are 1.9 and 4.5 per 100 000 person-years, respectively (Klaeboe *et al*, 2005). In the United States, the incidence rates with similar age-standardisation estimated from figures provided by the Central Brain Tumor Registry of the United States were 1.8 for men and 4.2 per 100 000 for women in 2006. Incidence of meningiomas varies depending on whether autopsies are included or not (Haddad *et al*, 2003).

Increasing incidence rates of meningiomas have been reported from several industrialised countries since the early 1980's (Christensen *et al*, 2003; Klaeboe *et al*, 2005), and the increase is most pronounced in older age groups (Maurice-Williams and Kitchen, 1993). The increase among the elderly can be explained by several factors. Indolent cases unrelated to the symptom for which the examination was conducted (eg, post-traumatic computerised tomography (CT)) are likely to be most common in older age groups. Also, introduction of new radiological techniques has allowed more non-invasive examinations for inoperable patients.

As meningiomas are benign in at least 9 out of 10 cases, they are not covered by most cancer registries. Nevertheless, in Finland, as in other Nordic countries, all physicians, pathologic laboratories and hospitals are obliged to report all tumours of the central nervous system, both malignant and benign, to the cancer registry.

The nationwide, population-based FCR has a practically complete coverage of solid cancer cases in Finland (Teppo *et al*, 1994). However, the registration of benign tumours of the central nervous system is not as complete as that of malignant neoplasms. Particularly, cases that are not treated surgically and lack histological confirmation are likely to be under-reported.

The aim of our study was to quantify undercount in the cancer registry and provide corrected estimates of meningioma incidence.

## Materials and methods

The meningioma cases were identified from Pirkanmaa Hospital District in Finland, which is the catchment area for Tampere University Hospital with a population of 447 051 in 2000. The study period was from November 2000 through June 2001 (eight months).

The cases were identified from four clinical data sources within Tampere University Hospital: (1) neurosurgical clinic; (2) pathology database; (3) hospital discharge database and (4) autopsy database. The department of neurosurgery provided a list of cases seen by their neurosurgeons, including operated and non-operated cases, outpatients and consultations on patients at other units. The pathology database included all cases diagnosed at the pathology unit (including biopsy). An autopsy database with diagnoses made in post-mortem examinations was also used. The hospital discharge database covers the major diagnoses of all in-patients admitted to the hospital.

The cases based on these clinical data sources and verified from the hospital records were compared with the case list of meningioma patients retrieved from the FCR. Information extracted from each source included the national unique personal identification number, place of residence, diagnosis, date of diagnosis and method of confirmation. Only residents in municipalities within the Pirkanmaa Hospital District were included. Only incident intracranial meningioma cases from November 2000 till June 2001 were included in this study.

The permission to obtain data from the FCR was granted by the National Research and Development Centre for Welfare and Health (STAKES). The study involved no contact with patients and was, therefore, exempt from a written informed consent in accordance with the Finnish regulations.

The world standard population was used in the age-standardisation (Segi, 1960). The incidence rates were calculated in 5-year age groups (even though presented in 10-year age groups in the tables). Confidence intervals (CIs) for the incidence rates were calculated assuming that the observed numbers of meningioma cases followed a Poisson distribution (Breslow and Day, 1987). CI for cancer registry coverage was estimated applying the general formula for the CI of a proportion.

## Results

Altogether, 42 incident intracranial meningioma patients were identified. The data sources from different hospitals identified a total of 39 patients and the FCR had information on 3 additional patients.

Altogether, the FCR covered 29 intracranial meningiomas during the study period. Of those, 27 fulfilled the inclusion criteria, whereas 2 were not incident cases, but recurrences.

Twenty-nine patients with newly diagnosed meningiomas were identified from the records of the neurosurgical department of Tampere University Hospital. Eighteen patients were operated on and 11 were treated conservatively. Twenty-six patients were identified from the Tampere University Hospital discharge database. Twenty-six patients fulfilling our definitions were found from the pathology database. Two of them were incidental cases detected in the 210 autopsies performed during the study period (Table 1).

Of the clinically recorded 39 patients, the FCR had registered 24. Of the 15 missing cases, 2 were found in the hospital discharge register only, based on radiological confirmation. No clinical or pathological cancer registry notifications were received at the FCR for 13 patients; 9 with a clinical diagnosis and 4 with a pathological verification.

The FCR had information on three patients not found in the clinical records: one was a histologically verified case and two were suspected meningiomas based on radiological findings only. All three were in-patients at the Tampere University Hospital, but without a discharge diagnosis of meningioma.

The cancer registry covered 27 of the 42 meningiomas (64%). The completeness of the FCR was 69% (95% CI, 55–83) of the 42 cases fulfilling the criteria, including the 2 recurrent cases.

**Figure 1:**
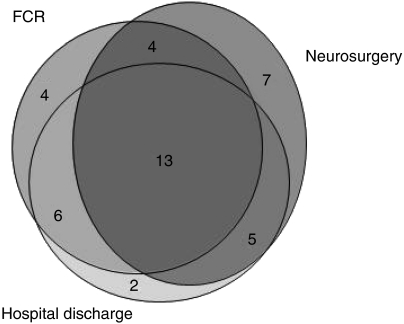
The numbers of meningioma cases from Pirkanmaa Hospital District in the Finnish Cancer Registry, Tampere University Hospital discharge registry and neurosurgical department patient list, November 2000 – June 2001. The area reflects the number of cases in each source and their overlap.

Of the 42 cases, only 11 (26%) were found in all the four data sources (autopsy database excluded). Only 13 (31%), including the previously mentioned 11 were covered by the three most comprehensive sources of information: neurosurgery department, hospital discharge database and FCR (Figure 1).

Diagnoses were histologically confirmed in 28 (67%) cases and based only on radiological finding (CT and/or MRI) in 14 (33%) cases. Under-registration was most common in cases aged 80 years and older (27%), as well as cases confirmed only radiologically (29%) (Table 1).

The age-standardised incidence rate of the cases from the FCR was 2.2 per 100 000 (95% CI, 0.3–4.1) for men and 9.6 (95% CI, 5.6–13.6) for women. The corresponding incidence rates for the best estimates were 2.9 per 100 000 person-years (95% CI, 0.7–5.0) for men, and 13.0 (95% CI, 8.7–17.3) for women.

## Discussion

None of the five sources including the cancer registry had a comprehensive coverage of meningioma cases. Completeness of the cancer registry was approximately two-thirds of all cases. The corrected incidence rates were a third higher than those reported by the cancer registry.

In our study, the corrected age-standardised incidence rates were 2.9 per 100 000 person-years for men, and 13.0 for women compared with 2.2 per 100 000 for men and 9.6 for women based on cancer registry data alone. The CIs (95%) for the incidence rates did, however, overlap, indicating that the estimates are compatible with each other. The study was limited by the small number of meningiomas. Nevertheless, bias such as under-registration is evaluated based on point estimates, not statistical significance. Despite this limitation, the study succeeded in identifying undercount in registration and providing corrected estimates of meningioma incidence.

There are several reasons for meningiomas not being notified to the FCR, such as asymptomatic meningiomas being followed clinically. In addition, incidental meningiomas, detected at autopsy may not be reported. However, only two meningiomas were found at autopsy in our study. This is less than anticipated, as the proportion of meningiomas in autopsies has been estimated to be as high as a quarter (Klaeboe *et al*, 2005). Also, incidental meningiomas were found in almost 1% of asymptomatic volunteers in a recent study (Vernooij *et al*, 2007).

Our results indicate that the incidence of meningiomas is considerably underestimated. We were able to obtain corrected incidence rates, which provide a more valid indication of the occurrence and disease burden than those based on a single data source. The findings also provide guidance for the conduct of not only occurrence studies, but also etiologic and prognostic studies, as they emphasise the need for case ascertainment and recruitment from several sources.

## Figures and Tables

**Table 1:** Number of meningioma cases by gender, age, hospital sources and diagnostic confirmation found and missing in the Finnish Cancer Registry

	**Finnish cancer registry**		
	**Yes**	**No**	**Total**
*Gender*			
Male	5	2	7
Female	22	13	35
			
*Age* (*years*)			
15–49	6	1	7
50–59	5	2	7
60–69	8	4	12
70–79	4	1	5
80–	3	8	11
			
*Hospital source*			
Pathology	22	4	26
Neurosurgery	17	12	29
Hospital discharge registry	19	7	26
			
*Diagnostic confirmation*			
Radiological	4	10	14
Microscopic^a^	23	5	28

a With or without radiological support for the diagnosis.
